# Impairment of object recognition, object location and spatial working memory performance in inbred RHA vs. RLA rats

**DOI:** 10.3389/fnbeh.2026.1837820

**Published:** 2026-06-25

**Authors:** Toni Cañete, Daniel Sampedro-Viana, Geison Souza Izídio, Berta Baró, Ignasi Oliveras, Cristóbal Río-Álamos, Margalida Coll-Andreu, Adolf Tobeña, Alberto Fernández-Teruel

**Affiliations:** 1 Medical Psychology Unit, Department of Psychiatry & Forensic Medicine, School of Medicine & Institute of Neurosciences, Autonomous University of Barcelona, Barcelona, Spain; 2 Behavioral Genetics Laboratory, Center for Biological Sciences, Department of Cell Biology, Embryology and Genetics, Federal University of Santa Catarina, Florianópolis, Brazil; 3 Department of Medicine, Faculty of Medicine and Health Sciences, International University of Catalunya, Barcelona, Spain; 4 Department of Psychology, School of Medicine, Austral University of Chile, Valdivia, Chile; 5 Department of Psychobiology and Methodology of Health Sciences, Institute of Neurosciences, Autonomous University of Barcelona, Barcelona, Spain

**Keywords:** cognitive deficits, long-term memory, object location, object recognition, RHA vs. RLA rats, spatial working memory, working memory

## Abstract

The inbred Roman rat strains, which have been selected and bred for their very high (RHA) or extremely poor (RLA) ability to acquire the two-way active avoidance task, constitute a bidirectional genetic model in which each strain differs from the other and/or from “standard” laboratory rat strains in a large number of behavioral and neurobiological phenotypes. Relative to Roman low-avoidance (RLA) rats, the Roman High-avoidance (RHA) rats exhibit increased mesolimbic dopamine function, social deficits, and impaired attention and cognitive flexibility, besides many other neurobehavioral phenotypes that make them a promising animal model of attention/cognitive-related syndromes, such as schizophrenia or attention deficit/hyperactivity disorder (ADHD). This study aimed to investigate non-spatial working memory and long-term memory in the Roman rat strains using the Object Recognition Test (ORT), a non-spatial declarative memory task that assesses object memory encoding, consolidation, and retrieval. The Object Location Test (OLT), which measures recognition of object location rather than differences between objects, was also used to evaluate short- and long-term memory. Between-strain spatial working memory differences were also evaluated using a delayed-matching-to-place (DMTP) task in the Morris water maze. Findings showed that RHA rats exhibited impaired working memory and long-term memory compared to RLA rats in the ORT. The degree of previous habituation to the testing context significantly affected object recognition memory in RHA rats. RHA rats also exhibited impaired short-term memory in the OLT. In the DMTP task, spatial working memory was also impaired in RHAs. The memory deficits shown by RHA rats seem to be compatible with their known (schizophrenia-like) alterations of the hippocampus and prefrontal cortex. The finding that relatively long habituation to the testing context rescues the long-term object-recognition memory deficit of RHA rats suggests that impaired attention, perhaps related to their relative hyperactivity, might be a factor underlying that deficit. The findings of the present study support the notion that the inbred RHA rat strain presents impairments in both non-spatial and spatial working/short-term memory, aligning with cognitive symptoms observed in patients with schizophrenia or with ADHD.

## 1 Introduction

The Roman rat strains have been selectively and bidirectionally bred for their very good (RHA; Roman High-avoidance) vs. extremely poor (RLA; Roman Low-avoidance) ability to acquire the two-way active avoidance task (Bignami, 1965), an approach-avoidance conflict that critically involves anxiety (Fernández-Teruel and McNaughton, 2023; Fernández-Teruel and Tobeña, 2020). As a consequence of such a bidirectional breeding, RHA and RLA rats differ in a wide range of neurobehavioral traits. Thus, RLA rats are more anxious/fearful and stress sensitive than RHAs (e.g., Fernández-Teruel et al., 2021; Fernández-Teruel et al., 2023; Giorgi et al., 2019; Steimer and Driscoll, 2003). On the other hand, in the context of the present study, relative to RLAs and other rat strains/stocks RHAs are characterized by increased impulsivity (Klein et al., 2014; Moreno et al., 2010), deficits in social behavior (Oliveras et al., 2022a; Sampedro-Viana et al., 2021, 2023a,b), novelty-induced hyper-locomotion, impaired attentional/cognitive abilities (Estanislau et al., 2013; López-Aumatell et al., 2009a,b; Martínez-Membrives et al., 2015; Tapias-Espinosa et al., 2018), and vulnerability to psychostimulant sensitization and drug addiction (Bellés et al., 2021, 2023; Dimiziani et al., 2019; Fernández-Teruel et al., 2021; Giorgi et al., 2019). Consistent with neurochemical/neuroanatomical phenotypes found in patients with schizophrenia, RHA rats also exhibit decreased function and volume of the medial prefrontal cortex (mPFC) and hippocampus (HPC) (Garcia-Falgueras et al., 2012; Meyza et al., 2009; Río-Álamos et al., 2019; Sallés et al., 2001; Tapias-Espinosa et al., 2019, 2023), increased functional tone of the mesolimbic dopamine system (Giorgi et al., 2019) and a dramatic deficit of central metabotropic glutamate-2 (mGlu2) receptors, along with increased density of 5-HT2A (serotonin 2A) receptors in the PFC (Elfving et al., 2019; Klein et al., 2014; Wood et al., 2017), alterations of several pre−/post-synaptic markers and increased density of pyramidal “thin” (immature) dendritic spines in the PFC (Elfving et al., 2019; Sánchez-González et al., 2021; Sønderstrup et al., 2023). The described neurobehavioral phenotypic profile of RHAs suggests that this strain can be considered as a promising animal model for studying some schizophrenia-relevant features along with vulnerability to drug addiction and even ADHD-related phenomena (see reviews by Białoń and Wąsik, 2022; Fernández-Teruel et al., 2021; Fernández-Teruel et al., 2023; Giorgi et al., 2019; Jones et al., 2011; Oliveras et al., 2023; Russell, 2011).

The inbred strains of RHA and RLA rats, derived from the Swiss RHA/Verh and RLA/Verh lines, were created and established at our laboratory in 1996. Studies in these Roman strains have revealed that inbred RHA rats are impaired at spatial reference learning/memory in the water maze (Martínez-Membrives et al., 2015; Río-Álamos et al., 2019), but findings on spatial working memory in inbred RHA vs. RLA rats have yielded either null or inconclusive results (Oliveras et al., 2015; Río-Álamos et al., 2019). Moreover, there is at present no information on the performance of the inbred Roman rat strains in non-spatial (and unconditioned) memory tasks, such as the object recognition test (ORT). Likewise, there has been no study on the performance of both inbred strains in the object location test (OLT), which involves spatial components, i.e., relocation of identical objects.

The ORT is considered a kind of non-spatial declarative memory task that involves components of episodic-like memory (Aggleton and Nelson, 2020; Amorós-Aguilar et al., 2020; Ennaceur and Delacour, 1988; Ennaceur et al., 1997). In the ORT rodents demonstrate a preference for exploring a novel object significantly more than a familiar one, which indicates that the memory of the familiar object was adequately encoded, consolidated, and subsequently retrieved to guide the rodent’s behavior during the test session (Blokland and Sesia, 2023; Cohen and Stackman, 2015; Ennaceur and Delacour, 1988; Si et al., 2023). The OLT, instead, measures how an animal recognizes that a given object has been moved to a different location, and thus it involves spatial processing. In both tasks, no training other than arena habituation is necessary to elicit object exploratory behavior (Aggleton and Nelson, 2020; Cohen and Stackman, 2015; Ennaceur et al., 1997).

The above reviewed neurobehavioral profiles, and the strain differences in several attentional/cognitive tasks (e.g., spatial reference learning/memory, latent inhibition, fear conditioning memory, impulsivity tasks), in which RHA rats are consistently impaired relative to RLAs (reviewed by Fernández-Teruel et al., 2021), prompted us to investigate the performance differences between both inbred strains regarding memory performance in the ORT, the OLT and the DMTP task. In line with the above evidence, our main hypothesis was that the RHA strain would exhibit worsened working/short-term memory and long-term memory performance relative to the RLA rat strain.

## 2 Materials and methods

### 2.1 Animals

A total of 181 male inbred Roman rats were used, comprising 92 RLA and 89 RHA rats. All rats were experimentally naive and came from the permanent breeding colonies maintained at our laboratory of the Autonomous University of Barcelona (Spain) since 1996. Mean body weights (± SD) at the beginning of the experimental procedures were 335.0 ± 37 g for study 1, 375.8 ± 30 g for Study 2, 384.3 ± 38 g for Study 3, and 449.5 ± 36 g for Study 4. They were housed in standard-size macrolon cages in same-sex pairs, under a 12:12 h light–dark cycle (lights on at 8:00 a.m.), with controlled temperature (22 ± 2 °C) and humidity (50–70%). Food and water were available ad libitum. Except when otherwise indicated, all experimental procedures were conducted between 9:00 and 13:30 h.

All procedures were carried out according to the protocols approved by the Animal Care and Use Committee of Autonomous University of Barcelona (CEEAH number 5022), with authorization from the Department of Environment of the Generalitat de Catalunya (number 9676), and with guidelines approved by the European Union Council Directive for the care and use of laboratory animals (2010/63/EU).

### 2.2 Study 1: object recognition test under two habituation schedules

**Figure 1:**
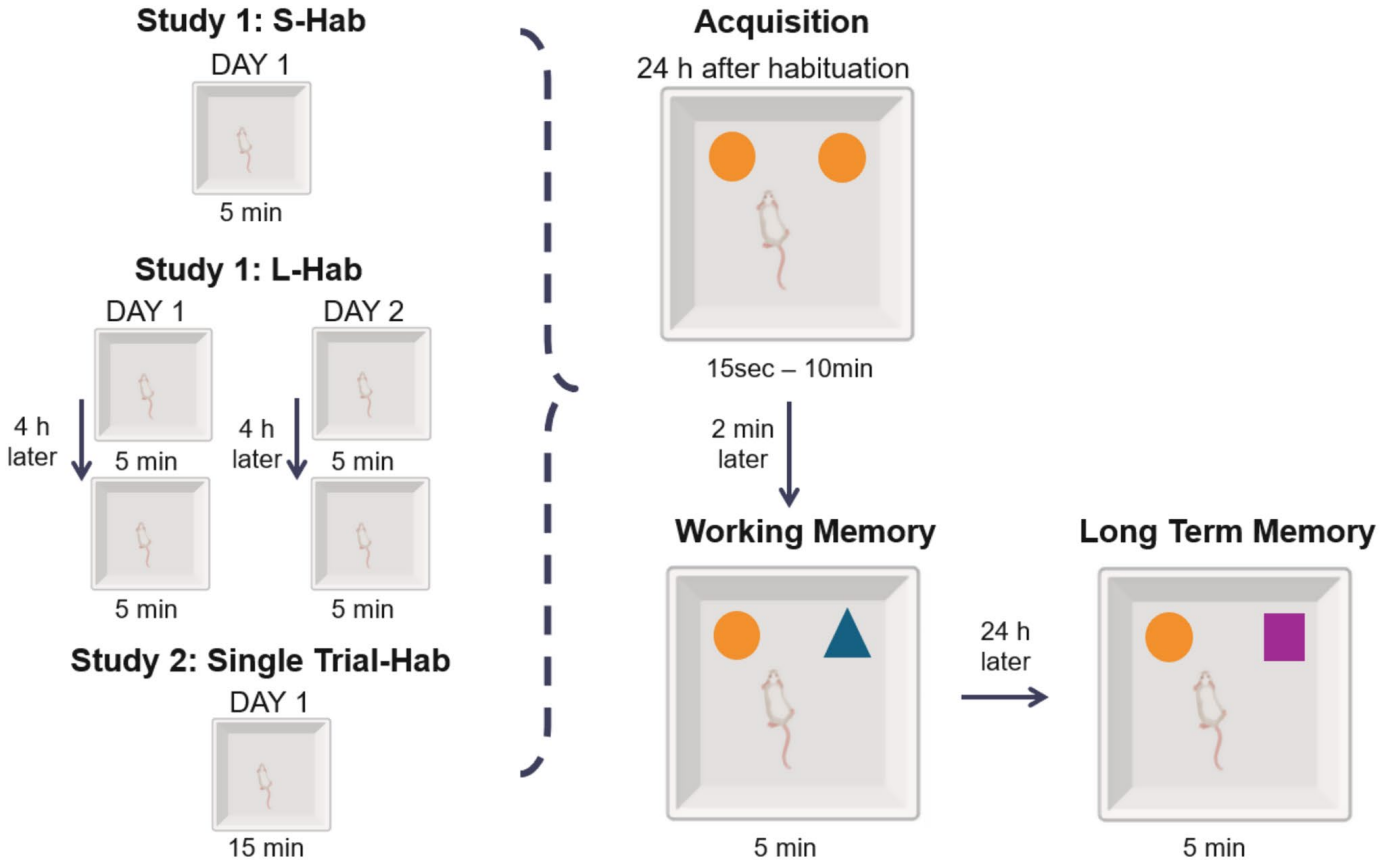
Procedure of the object recognition test (ORT) in studies 1–2, following long (L-Hab) or short (S-Hab) habituation (study 1), or 15-min single-trial habituation (Single Trial-Hab; study 2).

Twenty-four male RHA and 24 RLA rats were used. They were 3–4 months old. The ORT was conducted in a square wooden box (65 × 65 × 35 cm) painted white. Three objects were used: a Duplo^®^ Lego^®^ figure shaped like an inverted T, a Fanta^®^ (orange soda) can, and a blue plastic shelf wall bracket. The ORT procedure and the selection of objects were selected based on our previous study by Amorós-Aguilar et al. (2020), who showed that rats of the same strain and age had no spontaneous preference for any of these objects. Likewise, preliminary object preference testing confirmed here that the three objects elicited similar levels of exploration in both Roman rat strains combined (*n* = 24; means ranging from 10.7 to 12.6 s exploration in a 5-min trial), with no significant differences between the strains nor between the three objects. The objects were fixed to the floor of the box using double-sided adhesive tape, and illumination on the floor remained at 20 lux. Object positions and novel/familiar assignment were counterbalanced across animals. The ORT was conducted following one of two habituation protocols: the Long Habituation protocol (L-Hab), in which half of the animals received four 5-min (min) habituation sessions (two per day, morning and afternoon) before the test; or the Short Habituation protocol (S-Hab), consisting of a single 5-min session 1 day prior to testing (see Figure 1).

To confirm that habituation occurs with repeated exposure to the testing context (Estanislau et al., 2013; Guitart-Masip et al., 2008) the number of crossings and time spent self-grooming were measured during the first and fourth habituation sessions (in the L-Hab condition; see below) These sessions, as well as all subsequent testing trials, were recorded using a video camera mounted above the apparatus. Intermediate (2nd and 3rd) habituation sessions were not recorded. To prevent the presence of olfactory cues, both the objects and the experimental box were cleaned with a 70% ethanol solution in distilled water and dried before and after each trial. Visual cues remained fixed on the walls of the testing room throughout all sessions.

Twelve rats from each strain (RHA and RLA) underwent the Long Habituation protocol (L-Hab), and the remaining twelve from each strain underwent the Short Habituation protocol (S-Hab) prior to Object Recognition Testing for working memory and long-term memory (see below). Rats from each of the four experimental groups originated from at least 10 different litters. The ORT protocol is illustrated in Figure 1. Each session is described in detail below.

#### 2.2.1 Habituation

Rats were habituated to the testing environment either through one (S-Hab) or four (L-Hab; two per day, morning and afternoon) 5-min sessions, depending on group assignment. After each session, the animal was returned to its home cage, and the box was thoroughly cleaned with 70% ethanol and paper towels to eliminate potential olfactory cues (Figure 1). During the first (S-Hab and L-Hab conditions) and the fourth (L-Hab condition) habituation sessions, crossings (i.e., squares crossed) and time spent self-grooming were scored by a well-trained observer blind to group assignment.

#### 2.2.2 Acquisition trial

On the day following the habituation phase, animals underwent the acquisition trial (ACQ), during which they were required to accumulate at least 15 s (s) of object exploration. The trial ended when a rat achieved the 15-s criterion, and its maximum duration was 10 min. Only one rat, from the RHA S-Hab group, failed to meet the exploration criterion in 10 min and was excluded from subsequent testing (thus RHA S-Hab group *n* = 11). In this trial, two identical objects were placed side by side on the upper half of the box (Figure 1). Rats were placed in the box facing away from the objects and allowed to explore until they reached the criterion or the 10-min limit. Object exploration was defined as the animal directing its nose within at a distance ≤ 2 cm of the object; other behaviors, such as sitting on or resting against the object, were not considered exploration. After each acquisition trial, the animal was returned to its home cage for the 2-min inter-trial interval while the objects and the box were cleaned with 70% ethanol. Subsequently, the rat was placed back into the apparatus for the retention trial (Figure 1). The 15-s exploration threshold was chosen because shorter object exploration times may impair memory formation (Akkerman et al., 2012; Amorós-Aguilar et al., 2020).

#### 2.2.3 Retention trials

Two retention trials were conducted. The first, performed 2 min after the ACQ trial (2-min retention; working memory), presented the animal with one familiar object from the ACQ session and one novel object, placed side by side on the upper half of the box (Figure 1). The rat was placed in the box facing away from the objects and allowed 5 min to explore. After the trial, the box was cleaned with 70% ethanol and the animal returned to its home cage.

The second retention trial (24-h retention; long-term memory) was conducted 24 h (h) later. In this session, the previously presented familiar object was paired with a new novel object, again placed on the upper half of the box. Rats were introduced facing away from the objects and given a maximum of 5 min to explore. The box and objects were cleaned with 70% ethanol following each trial (Figure 1).

In all trials, object exploration was scored by a trained observer blind to group assignment.

The ORT relies on the natural tendency of rodents to explore novel over familiar objects. Therefore, two discrimination indexes were calculated: (1) The “time spent exploring the novel object minus the time spent exploring the familiar one” (Subtraction Index, SI). Values above zero are considered indicative of successful memory retrieval. Values close to zero suggest random exploration, interpreted as a failure to recall the familiar object (e.g., Cohen and Stackman, 2015; Ennaceur and Delacour, 1988). (2) The “time spent exploring the novel object divided by the total time spent exploring both objects x 100” (Discrimination Index, DI).

### 2.3 Study 2: object recognition task with longer (15-min) single-trial habituation

Thirteen male RHA and 14 RLA rats were used in this study. All animals were 3–4 months old and originated from at least 10 different litters per strain. The same three objects used in study 1 were employed here.

The procedure was identical to that described in study 1, with the exception that rats in Study 2 underwent a single 15-min habituation session conducted 24 h prior to the acquisition trial (Figure 1). Visual cues remained fixed on the walls of the testing room throughout all sessions. Object exploration was scored by a trained observer blind to group assignment.

### 2.4 Study 3: object location recognition task

**Figure 2:**
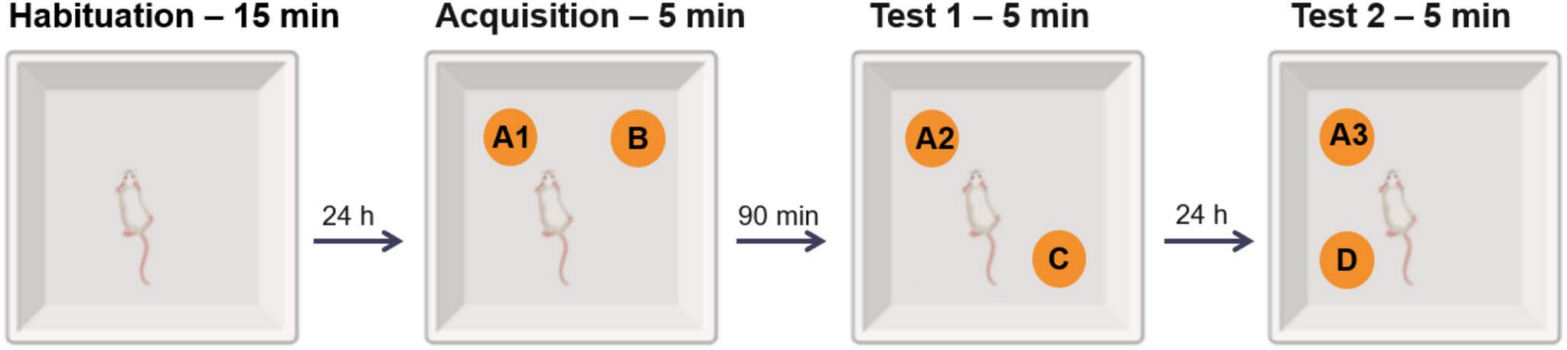
Procedure of the OLT (study 3). Two identical objects were used. The experimental timeline consisted of four sessions: Habituation; animals explored the empty arena for 15 min. Acquisition; 24 h later, rats explored two identical objects (A1 and B) for 5 min. Test 1 (Short-term memory); 90 min after acquisition, the object in (B) was moved to a novel location (C), and exploration was recorded for 5 min. Test 2 (Long-term memory); 24 h after acquisition, the same object was moved to the final corner (D) for a 5-min session. Gray arrows indicate the flow of the procedure, while letters (A1–A3, B–D) denote specific object positions used for the calculation of spatial discrimination savings.

Eleven male RHA and 10 RLA rats were used in this study. All animals were 3–4 months old and originated from at least 10 different litters per strain. The OLT, adapted from Velázquez et al. (2019), focused on the spatial relocation of familiar objects. The procedure consisted of four sessions conducted over two consecutive days: habituation, acquisition, Test 1 (90 min after acquisition, short-term memory), and Test 2 (24 h after acquisition, long-term memory) (Figure 2).

During habituation, animals were allowed to explore the empty arena for 15 min. In the acquisition session, conducted 24 h later, two identical objects (Fanta^®^ orange soda cans, 11.5 cm tall) were placed in adjacent corners of the arena, equidistant from the walls (10 cm), and rats explored them for 5 min. After a 90-min delay, Test 1 was conducted: one of the objects was moved to the diagonally opposite corner, and rats were reintroduced to the arena for 5 min to evaluate short-term memory. Twenty-four hours later, in Test 2, the same object was relocated to the remaining corner, and rats explored the arena for another 5 min to assess long-term memory. Visual cues remained fixed on the walls of the testing room throughout all sessions. To facilitate spatial orientation during the OLT, a black vertical strip (10 cm wide) was attached to the center of the wall that the animals faced when introduced into the experimental box to serve as a salient visual cue.

All sessions were recorded using an overhead video camera. Object exploration was scored by a trained observer blind to group assignment. Exploration was defined as the animal directing its nose at a distance ≤2 cm from the object or touching it with the nose or front paws; sitting on or leaning against the object was not considered exploratory behavior.

Besides the classical memory (Subtraction –SI- and Discrimination –DI-) indexes, as explained above (see Study 1, section 2.2), we calculated A2-A1 (time exploring A2 minus time exploring A1, an index of exploration of the unmoved object) and C-B (time exploring C minus time exploring B, an index of exploration of the moved object), to asses short-term memory of object location between Test 1 and the Acquisition trial.

Similarly, to asses long-term memory between Test 2 and Test 1 we calculated the subtractions “A3 minus A2” (A3-A2) and “D minus C” (D-C).

### 2.5 Study 4: delayed matching-to-place task of spatial working memory using the Morris water maze

**Figure 3:**
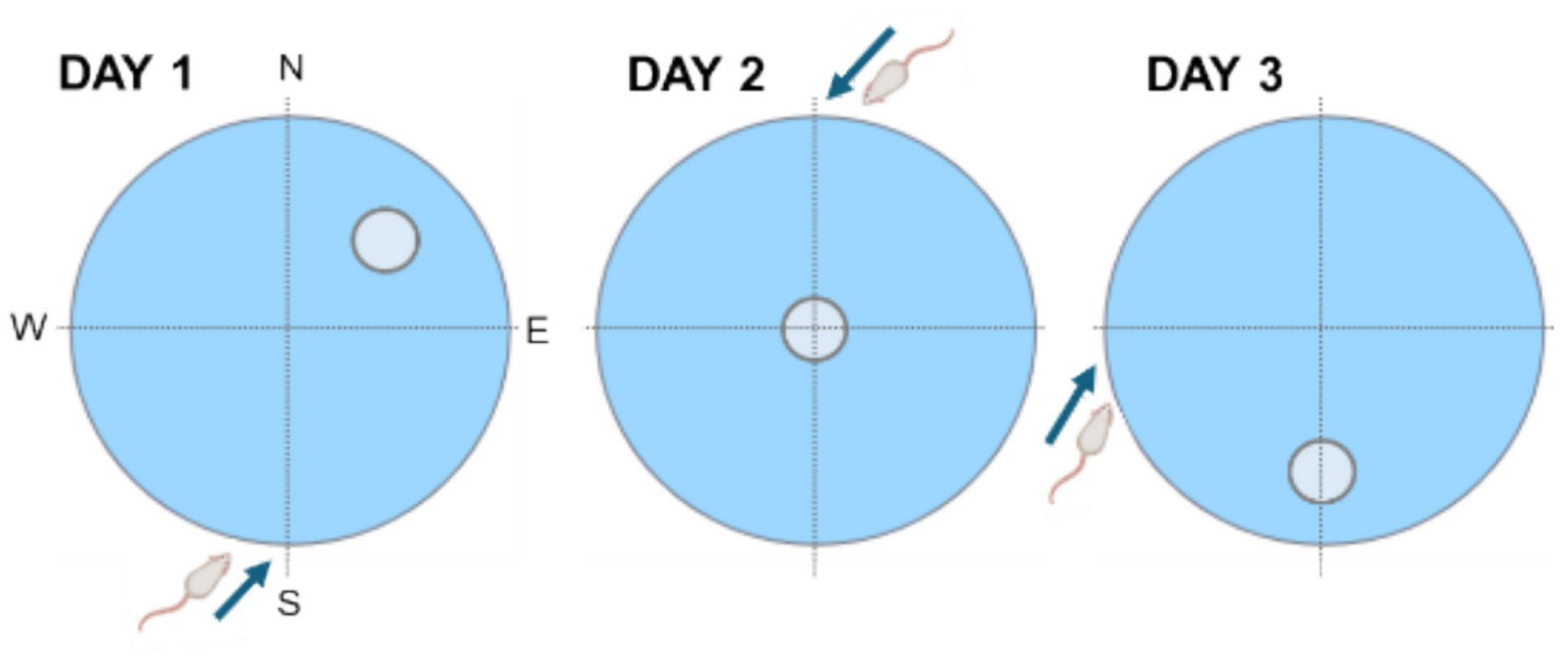
Procedure for the DMTP task (study 4). Arrows indicate the starting positions for both trials of each of the three training days in the DMTP (delayed-matching-to place; spatial working memory) task. Circles indicate the position of the submerged platform across the 3 days of training.

Forty-five male RLA and 40 RHA rats were used in this study. All animals were 5–6 months old and originated from 15 to 20 different litters per strain. The spatial working memory task was conducted in a circular pool (150 cm in diameter, 60 cm in height) filled with opaque water maintained at 24 °C to a depth of 30 cm. The delayed matching-to-place (DMTP) procedure was adapted from established protocols to index spatial working memory through average distance savings between the first and second trials (Whishaw, 1985). No proximal visual cues were available within the pool. Four starting positions (North, South, East, and West), evenly spaced around the perimeter, were designated for trial initiation, effectively dividing the tank into four quadrants (Figure 3).

A circular escape platform (15 cm in diameter, 28 cm in height) was submerged 2 cm below the water surface and located in one of the quadrants. Animal behavior was recorded using a ceiling-mounted video camera connected to a computerized tracking system (Smart v.2.5.14; PANLAB, Barcelona, Spain). The primary dependent variables were the latency (s) to find the hidden platform (and to jump onto it) and the total distance traveled before locating the hidden platform.

Each rat performed two trials per day across three consecutive testing days: a sample (acquisition) trial (T1) followed, after a 30-s delay, by a retention trial (T2). During the inter-trial interval, animals remained on the platform for 15 s and were then placed individually in a holding cage for another 15 s before the second trial began. Each trial began by placing the rat into the water facing the wall of the pool at one of the starting positions, and if the rat failed to reach the platform within 90 s it was gently guided to the platform by the experimenter. The starting position and platform location were pseudo-randomized across days but remained constant within each trial pair (Figure 3). The distal landmarks in the testing room consisted of several salient visual cues, including a blue water bucket, a dartboard, a foam baseball bat, and a white rectangular tray. These cues remained in fixed positions throughout the experimental sessions to provide stable spatial reference points for the DMTP task. Spatial working memory performance was indexed by “Mean T1–T2” calculated as the average difference in distance between T1 and T2 across the three testing days, and by the normalized (T1−T2)/(T1 + T2) index, also averaged for the three testing days.

### 2.6 Statistical analyses

All statistical analyses were performed using IBM SPSS Statistics (version 29.0.1.0), and all graphs were generated with GraphPad Prism (version 8.0.1).

Student’s *t*-tests for independent samples were applied to data (crossings and grooming) from the first habituation session in study 1. To evaluate within-subject changes during the L-Hab protocol, a repeated-measures ANOVA (2 strains × 2 habituation sessions) was conducted comparing crossings and grooming between the 1st and 4th habituation sessions.

To analyze retention performance in the ORT (study 1), a two-way ANOVA (strain × habituation protocol) with repeated measures (WM and LTM indexes) was applied. *Post hoc* comparisons were conducted using Duncan’s multiple range test, given our *a priori* hypotheses regarding strain differences in memory performance.

Data from study 2 were analyzed using Student’s *t*-tests for independent samples.

For the OLT (study 3), analyses included within-trial and between-trial comparisons of exploration time directed toward the moved versus unmoved object in Test 1 (90 min delay) and Test 2 (24 h delay), using either Student’s *t*-tests, for within test comparisons between the two rat strains, or repeated-measures ANOVA (2 strains x 2 object positions) and ANCOVA (2 strains x 2 object positions, taking the subtraction A2-A1 values as covariate; see Figure 2).

For the DMTP task, between-strain differences in spatial working memory performance were assessed by comparing the “Mean T1–T2” (average of the three “trial 1 minus trial 2” distance traveled to reach the hidden platform) across the three testing days using Student’s *t*-test for independent samples. The “(T1−T2)/(T1 + T2)” index was also analyzed using a Student’s *t*-test for independent samples.

Correlation analyses (Pearson’s *r*) were performed between acquisition time and retention indexes as a control measure to rule out potential exploratory biases. The absence of significant correlations confirms that memory performance was not a mere reflection of differences in object exploration during the acquisition trial, suggesting that the observed strain differences result from divergent mnemonic processes occurring after the acquisition phase.

Significance was set at *p* ≤ 0.05. One-tailed “p” was considered, when necessary, since we had the *a priori* directed hypotheses (based on previous findings on RHA vs. RLA differences in attentional/cognitive tests; see Introduction) that RHA rats would perform worse than RLAs in each of the memory tests.

## 3 Results

### 3.1 Study 1: object recognition test under two habituation schedules

#### 3.1.1 Habituation

The results of the habituation procedures are presented in Table 1. A Student’s *t*-test on the number of crossings during habituation session 1 (pooling all rats by strain) revealed a significant strain effect (t(45) = 3.15, *p* = 0.003), with RHA rats exhibiting more crossings than RLA rats. A similar analysis on grooming time also showed a significant strain effect (t(45) = 7.52, *p* < 0.001), indicating that RHA rats groomed less than RLA rats (see comparisons in Table 1).

**Table 1:** Results from the 1st and 4th sessions of context habituation (crossings and grooming) in the short (S-Hab) and long (L-Hab) habituation protocols (study 1).

Strain	Habituation protocol	n	Crossings Hab-session 1	Crossings Hab-session 4	Grooming Hab-session 1	Grooming Hab-session 4
RLA	S-Hab	12	60.7 ± 3.9		17.1 ± 2.3	
	L-Hab	12	68.5 ± 5.9	45.4 ± 6.5	20.1 ± 3.1	49.7 ± 7.6
RHA	S-Hab	11	85.3 ± 6.5*		1.8 ± 1.0*	
	L-Hab	12	84.9 ± 6.7*	70.2 ± 6.5	1.6 ± 1.1*	7.5 ± 1.6
Student *t*-test: habituation session 1			Strain effect: t(45) = 3.15, *p* = 0.003 (strains pooled)		Strain effect: t(45) = 7.52, *p* < 0.001 (strains pooled)	
F and *p*-value of repeated measures ANOVA (session 1 and session 4)			Strain effect: *F*(1, 22) = 9.87, *p* = 0.005 Session effect: *F*(1, 22) = 9.26, *p* = 0.006		Strain effect: *F*(1, 22) = 32.07, *p* < 0.001 Session effect: *F*(1, 22) = 44.37, *p* < 0.001 Session*Strain interaction: *F*(1, 22) = 19.73, *p* < 0.001	

Means (± SEM) of crossings and grooming during habituation sessions 1 and 4 in study 1. * *p* < 0.05, Duncan’s test with respect to corresponding RLA group. See correspondence of group symbols in “Material and methods.”

A repeated-measures ANOVA on data from the Long Habituation (L-Hab) protocol, comparing behavior in sessions 1 and 4, revealed significant strain (*F*(1, 22) = 9.87, *p* = 0.005) and session effects (*F*(1, 22) = 9.26, *p* = 0.006) on crossings (Table 1).

As for grooming, there were main effects of strain (*F*(1, 22) = 32.07, *p* < 0.001), session (*F*(1, 22) = 44.37, *p* < 0.001), and a strain*session interaction (*F*(1, 22) = 19.73, *p* < 0.001).

#### 3.1.2 Acquisition session (ACQ)

**Figure 4:**
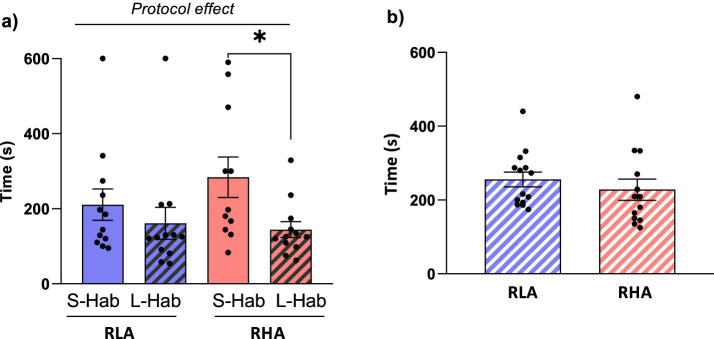
Differences in acquisition (ACQ) time (time needed to spend 15 s exploration of both sample objects) in the object recognition test (ORT) between RHA and RLA rats under **(a)** two habituation protocols (i.e., short, S-Hab; and long, L-Hab) in study 1, and **(b)** a 15 min single-trial habituation, in study 2. Means (± SEM) are shown. “Protocol effect” indicates a significant effect (ANOVA) of the habituation protocol. * *p* < 0.05 between the groups indicated (Duncan’s multiple range test). **(a)** Study 1: RLA S-Hab, *n* = 12; RLA L-Hab, *n* = 12; RHA S-Hab, *n* = 11; RHA L-Hab, *n* = 12. **(b)** Study 2: RLA, n = 14; RHA, *n* = 13.

A factorial ANOVA (2 strains × 2 habituation protocols) revealed a significant main effect of habituation protocol on acquisition time—defined as the time required to reach the 15-s exploration criterion (*F*(1, 43) = 5.32, *p* = 0.026), indicating that the L-Hab protocol globally improved acquisition performance, whereas such an improvement was more marked in RHA rats (see Duncan’s test in Figure 4a).

#### 3.1.3 Retention sessions (WM, LTM)

Absolute exploration times for the familiar and novel objects across all habituation protocols in Studies 1 and 2 are summarized in Table 2. Within group paired t-tests of time spent exploring the novel vs. the familiar object for the Short Habituation (S-Hab) protocol revealed a significant effect in both (2-min delay) working memory (*t*(11) = 3.99, *p* = 0.002) and (24 h delay) long-term memory (*t*(11) = 4.23, *p* = 0.001) sessions in RLA rats (Table 2). Similar effects were observed in the Long Habituation (L-Hab) protocol for both working memory (*t*(11) = 4.67, *p* = 0.001) and long-term memory (*t*(11) = 3.67, *p* = 0.004) trials in RLA rats. None of these comparisons was significant in RHA rats (Table 2).

**Table 2:** Total exploration time for familiar and novel objects in short (S-Hab) and long (L-Hab) habituation (study 1), and single-trial habituation (single trial-Hab; study 2) protocols.

Strain	Habituation protocol	*n*	Total time in the familiar object (WM)	Total time in the novel object (WM)	Total time in the familiar object (LTM)	Total time in the novel object (LTM)
RLA	S-Hab	12	11.0 ± 1.9	24.8 ± 2.8	11.7 ± 1.3	20.7 ± 1.8
	L-Hab	12	12.5 ± 1.9	21.2 ± 2.0	14.9 ± 0.9	23.5 ± 2.3
	Single trial-Hab	14	7.9 ± 1.2	14.5 ± 1.3	9.2 ± 1.2	11.3 ± 1.5
RHA	S-Hab	11	9.3 ± 1.8	11.0 ± 1.7	15.5 ± 1.7	15.1 ± 2.3
	L-Hab	12	9.7 ± 1.8	13.4 ± 2.9	14.0 ± 2.0	23.8 ± 3.9
	Single trial-Hab	13	13.1 ± 2.7	10.9 ± 1.2	13.2 ± 2.9	11.6 ± 1.0

Means (± SEM) are shown; *n*, number of rats/group. WM, LTM, working memory and long-term memory tests, respectively. **p < 0.01 ***p < 0.001 compared to the “Total time in the familiar object” in the same experimental group (paired t-tests).

“Acquisition time” was not correlated with any of the memory (SI, DI) indexes (all Pearson’s r < 0.15, all *p* > 0.30), indicating that the differences between RHA and RLA rats in the retention trials are independent of the time spent exploring the objects during the acquisition session.

**Figure 5:**
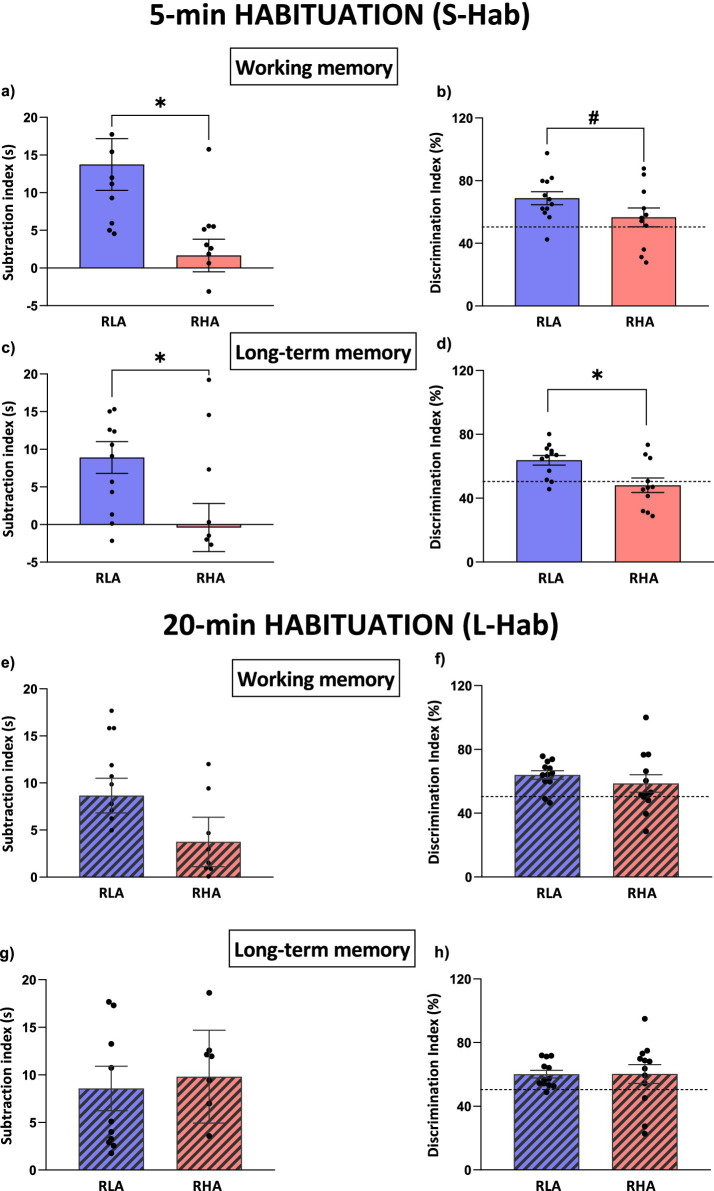
Results from study 1. Differences in “subtraction index” (SI) and “discrimination index” (DI) for working memory (WM) and long-term memory (LTM), in the ORT, between RHA and RLA rats under two habituation protocols (5-min, S-Hab; 20-min, L-Hab). **(a)** Means (± SEM) of SI for WM in the S-Hab protocol. **(b)** Means (± SEM) of DI for WM in the S-Hab protocol. **(c)** Means (± SEM) of SI for LTM in the S-Hab protocol. **(d)** Means (± SEM) of DI in LTM in the S-Hab protocol. **(e)** Means (± SEM) of SI for WM in the L-Hab protocol. **(f)** Means (± SEM) of DI for WM in the L-Hab protocol. **(g)** Means (± SEM) of SI for LTM in the L-Hab protocol. **(h)** Means (± SEM) of DI for LTM in the L-Hab protocol. * *p* < 0.05 between the groups indicated (Duncan’s multiple range test following significant ANOVA effects). #, *p* ≤ 0.03, one tailed, between the groups indicated (Duncan’s multiple range test following significant ANOVA effects). RLA S-Hab: *n* = 12; RLA L-Hab: *n* = 12; RHA S-Hab: *n* = 11; RHA L-Hab: *n* = 12.

The global ANOVA (2 strains × 2 habituation protocols × 2 retention trials) on SI revealed significant strain (*F*(1, 43) = 8.79, *p* = 0.005) and strain*habituation protocol (*F*(1, 43) = 4.38, *p* = 0.042) interaction effects, indicating that SI scores are globally higher in RLAs relative to RHA rats, whereas the RHA vs. RLA memory differences disappear under the L-Hab protocol (see Duncan’s test in Figure 5). The ANOVA on working memory SI index yielded a significant strain effect (*F*(1, 43) = 10.59, *p* = 0.002), as RLA rats exhibited better overall working memory performance than RHAs Figure 5). No significant ANOVA effects were found on the SI long-term memory index.

The global ANOVA (2 strains × 2 habituation protocols x 2 retention trials) on DI revealed significant strain (*F*(1, 43) = 8.32, *p* = 0.006) and marginal strain*habituation protocol (*F*(1, 43) = 3.86, *p* = 0.056) interaction, suggesting that DI scores are globally higher in RLAs relative to RHA rats, whereas the RHA vs. RLA memory difference disappear under the L-Hab protocol (see Duncan’s test in Figure 5). The ANOVA on DI working memory index showed a strain effect (*F*(1, 43) = 3.50, *p* = 0.034 one tailed), indicating better performance of RLA rats (Figure 5). Regarding the DI long-term memory index, there were strain (*F*(1, 43) = 3.49, *p* = 0.034 one tailed) and strain*habituation protocol (*F*(1, 43) = 3.53, *p* = 0.033 one tailed) interaction effects, indicating that DI scores are globally higher in RLAs relative to RHA rats, while the RHA vs. RLA difference in long-term memory disappears under the L-Hab protocol (see Duncan’s test in Figure 5).

### 3.2 Study 2: object recognition task with longer (15-min) single-trial habituation

**Figure 6:**
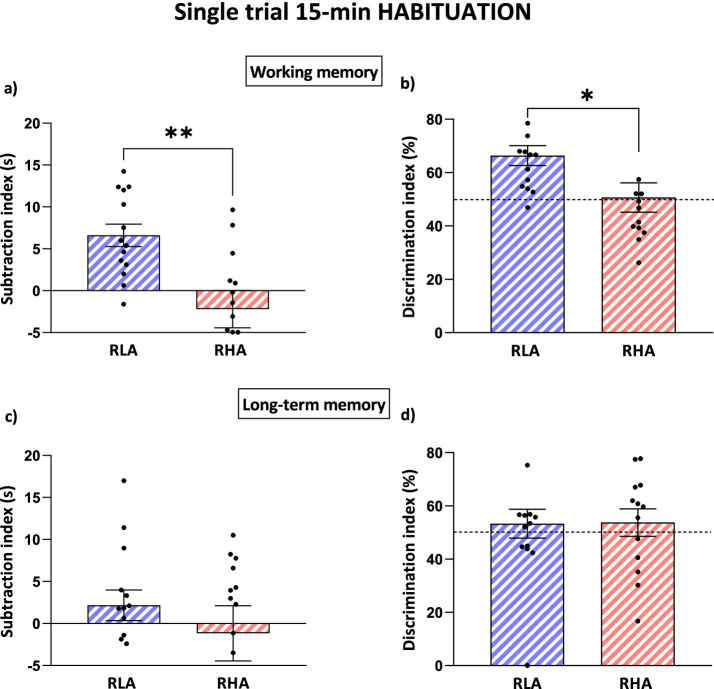
Differences in acquisition (ACQ) time (time needed to spend 15 s exploration of both sample objects) in the object recognition test (ORT) between RHA and RLA rats under **(a)** two habituation protocols (i.e., short, S-Hab; and long, L-Hab) in study 1, and **(b)** a 15 min single-trial habituation, in study 2. Means (± SEM) are shown. “Protocol effect” indicates a significant effect (ANOVA) of the habituation protocol. * *p* < 0.05 between the groups indicated (Duncan’s multiple range test). **(a)** Study 1: RLA S-Hab, *n* = 12; RLA L-Hab, *n* = 12; RHA S-Hab, *n* = 11; RHA L-Hab, *n* = 12. **(b)** Study 2: RLA, *n* = 14; RHA, *n* = 13.

No strain differences were found on “acquisition time” (t(25) = 0.80, *p* = 0.43; Figure 4b). The analysis of the ORT working memory trial (i.e., 2-min retention interval) yielded significant strain differences for both SI (t(25) = 3.47, *p* = 0.002) and DI (t(25) = 2.39, *p* = 0.025) variables, as RLA rats outperformed RHAs in both memory indexes (Figure 6). Within group paired *t*-tests of the time spent exploring the novel vs. the familiar object revealed a significant effect only in RLA rats during the 2-min retention trial (*t*(13) = 4.98, *p* < 0.001) (Table 2). Conversely, there were no strain differences in SI or DI variables in long-term (i.e., 24-h retention) recognition memory (*t*(25) = 0.91, *p* = 0.37, and t(25) = 0.06, *p* = 0.96, respectively) (Figure 6).

### 3.3 Study 3: object location task

**Figure 7:**
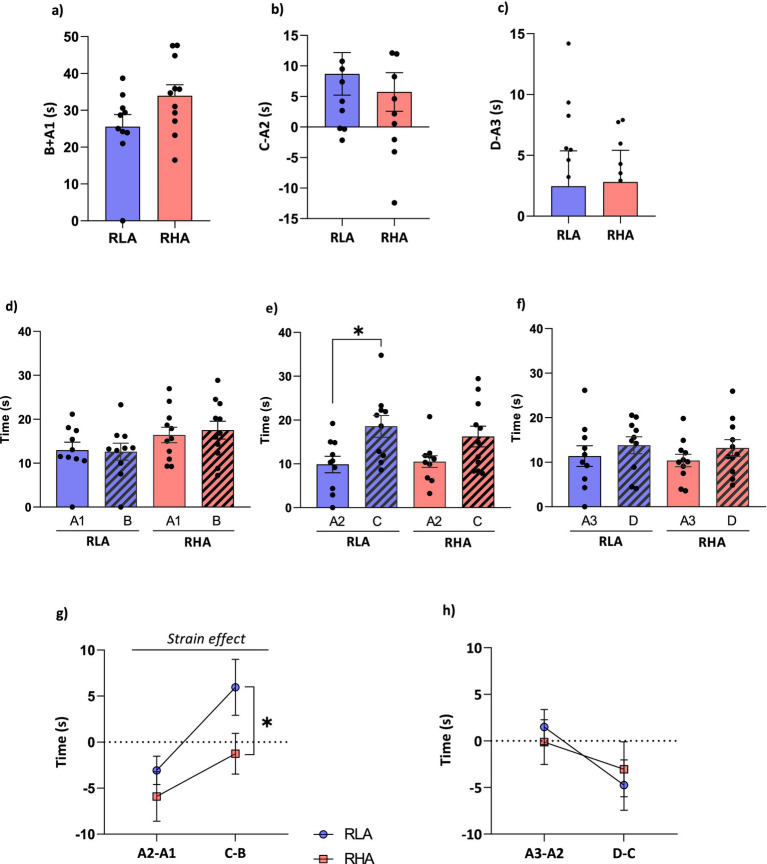
Object location test (OLT) results (study 3). **(a)** Mean ± SEM of total B + A1 exploration time (i.e., exploration of both objects) during the 5-min acquisition trial (no statistically significant difference, as expected). **(b)** Mean ± SEM exploration time of C–A2 (subtraction) object positions during the short-term memory (90 min retention delay) phase. **(c)** Mean ± SEM of exploration time of D–A3 (subtraction) object positions during the long-term memory (24 h retention delay) phase. **(d)** Mean ± SEM of total A1 and B exploration time during the 5-min acquisition trial (no statistically significant difference, as expected). **(e)** Mean ± SEM of total A2 and C exploration time during the 5-min Test 1, *p  < 0.05 between the objects indicated for RLA strain (Student’s t-test for paired samples). **(f)** Mean ± SEM of total A3 and D exploration time during the 5-min Test 2 (no statistically significant difference, as expected). **(g)** Mean ± SEM of A2–A1 and C–B difference scores for RLA and RHA groups. These indices reflect short-term memory of object location, by showing that rats of both strains devote the same exploration time to the unmoved object (A2–A1 index, Test 1 vs. Acquisition) while RLA rats show enhanced exploration of the moved object (C vs. B; C–B index; Test 1 vs. Acquisition). **(h)** Mean ± SEM of A3–A2 and D–C difference scores (i.e., Test 2 vs. Test 1) for RLA and RHA groups. “Strain effect”; indicates a significant (*p* < 0.05) strain effect from ANOVA with repeated measures (see details in main text). *p  < 0.033, Student’s t-test (one-tailed) of the C-B difference. RLA: *n* = 10, RHA: *n* = 11.

There were no strain differences in total exploration of both objects during the acquisition trial of the OLT (t(19) = 1.88, *p* = 0.075; Figure 7a).

Analysis of the usual within-trial SI measure in the OLT task did not yield significant strain differences in either test 1 (short-term memory) or test 2 (long-term memory) (t(19) = 0.63, *p* = 0.54, and t(19) = 0.09, *p* = 0.93, respectively) (Figures 7b,c). Analyses of the DI measure yielded similar nonsignificant results (not shown). Figures 7d,f show that object exploration times across the three sessions (acquisition, test 1 and test 2) were equal between both rat strains. The RLA strain showed a significant difference in exploration time during the 5-min test 1 trial, with significantly more time spent exploring object C than object A2 (paired *t*-test, t(9) = −2.49, *p* = 0.034; Figure 7e), thus suggesting some discrimination of the moved object.

Comparison of “time exploring C minus time exploring B” (C-B, Figure 7d) vs. “time exploring A2 minus time exploring A1” (A2-A1, Figure 7g) through an ANOVA with repeated measures (A2-A1 and C-B) yielded a global strain effect (*F*(1, 19) = 4.52, *p* = 0.047). This was confirmed by an ANCOVA of C-B, taking A2-A1 values as covariate (Pearson correlation between C-B and A2-A1, r = 0.01, *p* = 0.95; ANCOVA, *F*(1, 19) = 3.66, *p* = 0.036 one-tailed). Both the results of the ANOVA with repeated measures and the ANCOVA reflect the fact that RHA show a lower “C-B” difference than RLA rats (Student’s *t*-test for C-B, t(19) = 1.94, *p* < 0.033 one-tailed; Figure 7g), indicating impaired short-term object location memory in RHAs. In fact, only RLA rats showed positive discrimination of the moved object (C vs. B), as indicated by a mean group value clearly above “0” of the C-B index (Figure 7g).

No differences appeared between both rat strains regarding long-term memory when comparing the corresponding variables, i.e., “time exploring D minus time exploring C” (D-C) vs. “time exploring A3 minus time exploring A2” (A3-A2). The ANOVA with repeated measures (D-C and A3-A2) yielded non-significant effects of strain (*F*(1, 19) = 0.45, *p* = 0.60) (Figure 7h).

### 3.4 Study 4: delayed matching-to-place task of spatial working memory

**Figure 8:**
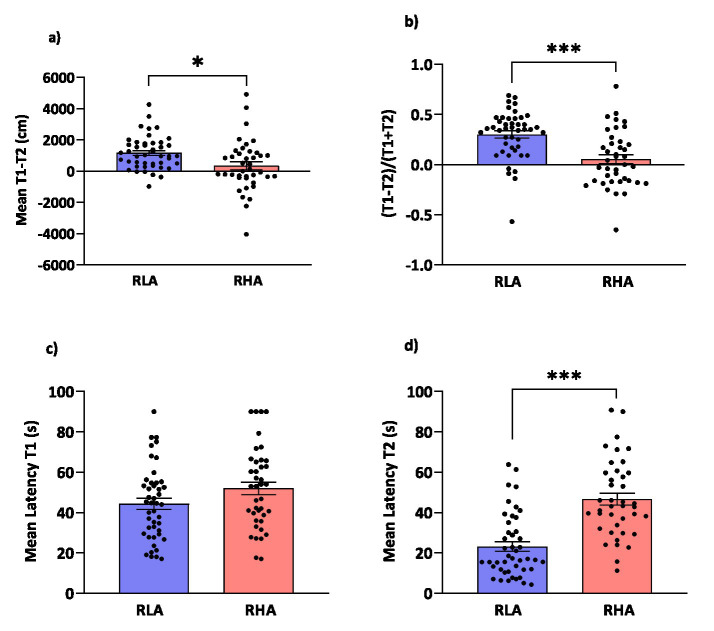
Results from study 4 in the DMTP (delayed matching-to-place) task in the Morris water maze. **(a)** Means (± SEM) of the average “(Trial 1 – Trial 2)/3 days” distance (cm) differences between the RHA and RLA rat strains. **(b)** Means (± SEM) of the memory index, calculated using the normalized formula (T1−T2)/(T1 + T2) averaged for the 3 training days. **(c)** Means (± SEM) of the average latency (s) to reach the platform during Trial 1 (sum of T1 latencies/3 days) in the DMTP task. **(d)** Means (± SEM) of the average latency (s) to reach the platform during Trial 2 (sum of T2 latencies/3 days) in the DMTP task. ^*^p < 0.025 between both groups. ^***^ *p* < 0.001 between both groups. RLA: *n* = 45, RHA: *n* = 40.

Student’s *t*-test for independent samples revealed that the “mean T1–T2” (i.e., average distance savings between trial 1 minus trial 2 across the 3 training days) was significantly greater in RLA than RHA rats (t(83) = 2.30, *p* = 0.024; Figure 8a).

Similarly, the normalized learning index, calculated as (T1−T2)/(T1 + T2), was significantly higher in the RLA strain than in RHA rats (t(83) = 4.35, *p* < 0.001; Figure 8b), indicating superior spatial working memory and more efficient short-term savings in the RLA group (Figure 8b).

Finally, regarding latencies, no significant differences between strains were found in Trial 1 (t(83) = −1.86, *p* = 0.066; Figure 8c), although RLA rats showed significantly shorter latencies to reach the platform in Trial 2 compared to RHA rats (t(83) = −6.26, *p* < 0.001; Figure 8d).

## 4 Discussion

In the present study, we evaluated non-spatial working memory and long-term memory in two procedures of non-spatial object recognition (episodic memory; ORT) in the inbred Roman rat strains. The only related precedents that explored non-spatial working memory in the ORT were the studies by Delacour’s group (Guenaire and Delacour, 1985; Willig et al., 1991a,b, 1992), who for over a decade (1978–1992) maintained a French colony (at the Center de Recherches Roussel-Uclaf, Roussel-Uclaf, Romainville, France) of “outbred” Roman rat sub-lines that were the descendants of breeders obtained from the laboratory of Prof. Broadhurst (Birmingham, England). These authors carried out the ORT, showing that their outbred RHA rats exhibited worsened working (object recognition) memory at 1-min and 60-min retention intervals (Guenaire and Delacour, 1985; Willig et al., 1991a,b, 1992). In contrast, our Roman rats are “inbred” strains which were derived through brother/sister mating, starting in 1996, from breeders of the Swiss Roman/Verh sub-lines (maintained by Prof. Peter Driscoll, from 1972 to 2004, at the Eidgenössische Technische Hochschule-ETH, Zürich, Switzerland) (Fernández-Teruel et al., 2021). It was therefore necessary to explore whether our “inbred” Roman rat strains present differences in non-spatial working and long-term memory.

The present studies 1–2 show significant differences between RHA and RLA rats regarding their performance in the ORT. Specifically: (i) In study 1, RHA rats exhibited poorer non-spatial working memory performance in the ORT, as well as impaired long-term memory in the S-Hab condition, compared with their RLA counterparts. (ii) Remarkably, the memory deficits of RHA rats were restricted to conditions of short habituation (S-Hab), as longer arena habituation (L-Hab condition) rescued these impairments. (iii) Consistent with our previous studies with open field-like tests, during the initial exposure to the testing box in study 1, RHA rats showed more activity (crossings) and lower self-grooming than RLA rats (Díaz-Morán et al., 2012; Estanislau et al., 2013; López-Aumatell et al., 2009a; Tapias-Espinosa et al., 2018). (iv) During the habituation phase preceding the ORT, the animals in the L-Hab condition exhibited an increase of grooming and a decrease of crossings between sessions 1 and 4, consistent with habituation learning occurring during repeated exposure to the testing context (Estanislau et al., 2013; Fernández-Teruel and Estanislau, 2016; Tapias-Espinosa et al., 2018). (v) In study 2, in which a single but relatively longer (15 min) habituation session was used, RHA rats displayed impaired working (object recognition) memory but again both rat strains did not differ in long-term memory.

In both studies with the ORT the inbred RLA strain displays better working (object recognition) memory than RHA rats, which is overall in agreement with the results observed between the French “outbred” Roman rat sublines in the 80s–90s (Guenaire and Delacour, 1985; Willig et al., 1991a,b, 1992). It should be noted that while 1–5-min retention intervals in the ORT are common delays used to evaluate memory (e.g., Akkerman et al., 2015; Antunes and Biala, 2012; Cohen and Stackman, 2015; Ennaceur and Delacour, 1988; Willig et al., 1991b, 1992), these time windows may recruit not only working memory but also short-term memory mechanisms. Therefore, our results regarding this task should be interpreted within the broader context of short-term mnemonic performance, as the 2-min delay does not strictly isolate working memory from other memory processes (e.g., a 2-min delay may still involve active acquisition processes overlapping with early consolidation processes; Akkerman et al., 2015). However, studies 1–2 further add that object recognition memory and between-strain differences depend on the degree of previous habituation to the testing context, as we have found that in our inbred Roman strains the working/short-term memory (in study 1) and long-term memory differences (in studies 1–2) disappeared after relatively longer habituation periods (i.e., 20 min in study 1 or 15 min in study 2). The results from study 1, in particular, suggest that when the inbred RHA rats are allowed to get enough knowledge about the testing context, later on they may better focus their attention (see below) on the objects –rather than on the surrounding context- and so their object memory is improved. This notion is in accordance with findings indicating that inbred RHA rats exhibit attentional deficits relative to RLA rats (Esnal et al., 2016; Fernández-Teruel et al., 2006; Fernández-Teruel et al., 2021; Fernández-Teruel et al., 2023; Moreno et al., 2010). In this context, it could be argued that the differential performance under short habituation might be attributed to strain differences in anxiety. However, it is well-established that RLA rats are the more anxious and stress-sensitive strain compared to the hypoemotional RHAs (Fernández-Teruel et al., 2021; Fernández-Teruel et al., 2023; Giorgi et al., 2019; Steimer and Driscoll, 2003). Since the more anxious RLA rats successfully discriminate objects even with minimal habituation, it is unlikely that anxiety is the factor impairing RHA performance. Instead, the rescue of the memory deficit by longer habituation in RHA rats suggests that their relative hyperactivity and attentional deficits, rather than anxiety, impair the initial encoding of object features in a novel environment. Once the context becomes familiar, these “distractions” diminish, likely allowing RHA rats to focus sufficient attention on the objects to form a stable memory.

We also studied whether RHA and RLA rats differ in the OLT (study 3), following our previously published procedure (Velázquez et al., 2019). The memory measures within the retention tests (i.e., comparison of object exploration times within each retention test) did not show significant differences. Nevertheless, when comparing the difference between the exploration time devoted to the relocated object in “Test 1 minus Acquisition session” (i.e., C-B) vs. the exploration difference of the fixed object in “Test 1 minus Acquisition session” (i.e., A2-A1), the C-B index was positive in RLA rats (i.e., they explored more the object C in Test 1 than in its initial position in the acquisition trial –object B-) and it was different from the negative value of C-B observed in RHA rats (see Figure 7g). Conversely, for the A2-A1 index (i.e., exploration of the unmoved object in both the “acquisition” and “Test 1” trials) there were no differences between the strains. Hence, such a significant difference in the C-B index indicates that RHA rats were impaired at detection/exploration of the relocated object, suggesting poorer short-term memory in RHAs than RLA rats.

Previous studies comparing males from both inbred Roman strains in the spatial delayed matching-to-place (DMTP) task yielded null or inconclusive results (Oliveras et al., 2015; Río-Álamos et al., 2019). That is why we have evaluated both rat strains in the DMTP task here using a much larger sample of males from each strain than the samples used in the above-mentioned studies. The results of this study (study 4) clearly show that RLA rats outperformed RHAs in the DMTP task, which assesses spatial working memory (Morris et al., 1986; Whishaw, 1985; Whishaw et al., 1995).

The present findings constitute an addition to the impaired performance of the inbred RHA strain (relative to inbred RLAs), and their outbred RHA/Verh ancestors (relative to their RLA/Verh counterparts), in other cognitive tasks, such as spatial reference (and reversal) learning (e.g., Escorihuela et al., 1995; Martínez-Membrives et al., 2015; Nil and Bättig, 1981; Río-Álamos et al., 2019), retention of fear conditioning (López-Aumatell et al., 2009a,b), habituation of the acoustic startle response (Oliveras et al., 2022b,c) and latent inhibition (Esnal et al., 2016; Fernández-Teruel et al., 2006), which suggest a global cognitive deficit of inbred RHA rats (Fernández-Teruel et al., 2021; Fernández-Teruel et al., 2023; Giorgi et al., 2019).

Both the medial prefrontal cortex (mPFC) and the hippocampus (HPC), and the interaction between them, have been proposed to be critically involved in different aspects of spatial short-term and working memory in laboratory rodents (Babl and Sigurdsson, 2025; Funahashi, 2017; McNaughton and Gray, 2024; Vogel et al., 2022; Wirt and Hyman, 2017), whereas there seems to be agreement that the perirhinal cortex is the main region required for non-spatial object recognition memory (Aggleton and Nelson, 2020; Cohen and Stackman, 2015). Under some conditions and retention intervals there seems, however, that the HPC (in interaction with the perirhinal cortex) may also play an important role in object recognition memory. In fact, Cinalli et al. (2020) have reported that the HPC supports strong object memory, while the perirhinal cortex supports weak object memory (Aggleton and Nelson, 2020; Cinalli et al., 2020; Cohen and Stackman, 2015). Object recognition memory may depend on the pure recognition of the stimulus (object) regardless of the context, or be based on associations made between the stimulus and its spatial location (object-place memory), between a stimulus and context in which it was encountered (object-context memory), or both (object-place-context memory) (see, e.g., Barker and Warburton, 2020). Recent evidence suggests that pure object recognition memory involves intervention of the perirhinal cortex (e.g., Barker and Warburton, 2020; Kim et al., 2014), whereas HPC, mPFC and perirhinal cortex involvement are all important for object-place-context memory, i.e., when associations of the stimulus (object) with contexts are important (Barker and Warburton, 2020). In sum, what the evidence suggests is the importance of a HPC-mPFC-perirhinal cortex network in forming associations between objects and the contexts they are encountered in (see Barker and Warburton, 2020, and references therein).

In this regard, it is interesting that, relative to RLAs, the inbred RHA rats have been shown to present lowered task-induced activity (as measured by c-Fos expression) and volume of the mPFC, as well as higher density of immature pyramidal dendritic spines (Río-Álamos et al., 2019; Sánchez-González et al., 2021; Tapias-Espinosa et al., 2019), a reduction of parvalbumin-expressing (GABAergic) neurons (Tapias-Espinosa et al., 2023) and an immature gene-expression profile in that region (Elfving et al., 2019; Sønderstrup et al., 2023). Likewise, RHA rats exhibit lowered pyramidal neuron density in the HPC, as well as reduced task-induced activation and volume of this region (Garcia-Falgueras et al., 2012; Meyza et al., 2009; Río-Álamos et al., 2017, 2019; Sallés et al., 2001). Collectively, the accumulated evidence points to a lowered function of the mPFC and HPC in inbred RHA rats compared with their RLA counterparts (Fernández-Teruel et al., 2021), which may underlie their impaired memory performance observed in various (spatial and non-spatial) tasks. Nevertheless, there has been no study on perirhinal cortex in RHA vs. RLA rats; thus, the relationship of perirhinal cortex function with the observed between-strain differences in the different memory tasks are issues that warrant further research.

Impairments of attentional processes and enhanced impulsivity (i.e., impaired top-down inhibitory control) in RHA rats may also be factors contributing to their impaired cognitive performance in spatial and non-spatial memory tasks (Bellés et al., 2021, 2023; Esnal et al., 2016; Fernández-Teruel et al., 2006; Klein et al., 2014; Moreno et al., 2010). Alterations of attention and impulsivity may arise from anomalies in the functional neuroanatomy of RHA rats, such as the aforementioned diminished function and abnormal maturation of the mPFC and HPC (Fernández-Teruel et al., 2021). Besides their involvement on spatial and episodic memory, both the mPFC and the HPC are crucial regions for top-down inhibitory behavioral control (e.g., Fernández-Teruel and McNaughton, 2023; Fernández-Teruel and Tobeña, 2020; McNaughton and Gray, 2024). Thus, these attentional-impulsivity profiles, possibly related to the impaired frontocortical-hippocampal maturation of inbred RHA rats (Fernández-Teruel et al., 2021), might at least partly underlie their memory deficits.

The finding that the worsened object recognition memory of RHA rats in the ORT under the S-Hab condition is reversed by longer habituation to the context (L-Hab condition in study 1, or 15-min habituation in study 2) may support the above contention. This suggests that when the context becomes more familiar due to longer habituation it favors more focused attention on the objects (i.e., familiar object vs. novel object discrimination), particularly in inbred RHA rats. This idea is also supported by the finding that, during the acquisition trial of study 1, achievement of the criterion of object exploration was improved under the L-Hab condition only in RHA rats. Thus, the lack of object recognition deficit under the L-Hab condition in RHA rats may be due to lowered “distraction,” to the decrease of other exploratory behaviors (such as the reduction in the number of crossings in the L-Hab condition) and/or diminished emotional arousal to the well-habituated context. In fact, variation of emotional arousal in the ORT, by using different context-habituation conditions, has been shown to markedly affect the effects of stress hormones and other drugs on object recognition memory in rats (e.g., Campolongo et al., 2013; Okuda et al., 2004). Furthermore, it is likely that the excess of context exploratory behavior (e.g., crossings; locomotor activity) of RHA rats is a factor that interferes with attention to the objects, as suggested by the fact that under the L-Hab condition (study 1) crossing numbers are reduced by the 4th habituation session, and this is paralleled by an improvement in both object exploration during the acquisition trial and object recognition memory in the RHA strain.

Habituation to novel contexts in rodents has been traditionally linked to hippocampal function (see review by Leussis and Bolivar, 2006). Hence, it is tempting to speculate that in RHA rats, which, as said earlier, have a less functional HPC (compared with RLA rats), the relatively weak (or incomplete) long-term contextual memory storage (due to short habituation, S-Hab) by the HPC would interfere with memory of the familiar object (i.e., familiar object recognition) in the S-Hab condition. Conversely, greater familiarity with the context (i.e., stronger memory of the testing context due to longer habituation) would free up the HPC to get more focused on memory storage of the familiar object, which would finally lead to better novel vs. familiar object discrimination.

The present studies have some limitations that should be acknowledged. Due to space and breeding restrictions (since we had to keep the females for breeding purposes in order to maintain our colony of Roman rats), it was not possible to include females in the present studies. When females and males have been compared in different tests, gender effects and strain x gender interactions have often been observed (Oliveras et al., 2022a,c). Therefore, it would be relevant to include females of both rat strains in future studies to explore the generalizability of the present findings. On the other hand, in spite of the consistent neural and molecular findings in the inbred RLA vs. RHA strains reviewed above, the neurobiological interpretations regarding prefrontal-perirhinal-hippocampal circuitry and their relation with the present cognitive results remain speculative in the absence of direct neural or molecular data. Thus, in order to establish neural-behavioral associations, and to strengthen translational validity, it would be desirable for future studies to combine behavioral and neurobiological approaches, such as we did in previous studies (e.g., Klein et al., 2014; Moreno et al., 2010; Peralta-Vallejo et al., 2025; Río-Álamos et al., 2017; Tapias-Espinosa et al., 2019).

The utility of RHA rats as a translational model for schizophrenia research is supported by several validation processes. Its face validity is evidenced by behavioral endophenotypes such as deficits in prepulse inhibition (PPI) and latent inhibition, hyperactivity and reduced social interaction (e.g., Esnal et al., 2016; Tapias-Espinosa et al., 2018, 2019, 2023; Sampedro-Viana et al., 2021, 2023a). The model’s construct validity is established by neurobiological markers that parallel the human condition, including reduced volume and function of the medial prefrontal cortex and hippocampus, increased functional tone of the mesolimbic dopaminergic pathway and (likely) reduced function of the mesocortical dopaminergic system, as well as a significant deficit in mGlu2 receptors and increased 5-HT2A receptor density, among other (e.g., Elfving et al., 2019; Giorgi et al., 2019; Fernández-Teruel et al., 2021; Klein et al., 2014; Meyza et al., 2009; Río-Álamos et al., 2019; Tapias-Espinosa et al., 2019; Wood et al., 2017). Finally, its predictive validity has been demonstrated through the reversal or attenuation of several of these behavioral impairments by both typical and atypical antipsychotic drugs, and oxytocin, as well as by the behavioral impairments induced by several pro-psychotic drugs (Oliveras et al., 2017; Sampedro-Viana et al., 2023b, 2024; Tapias-Espinosa et al., 2021). By reporting impairments in non-spatial and spatial short-term/working memory (that are rescued by context habituation under some conditions), the present study further expands the cognitive characterization of the RHA rat strain, and strengthens its translational validity for the study of some schizophrenia-relevant cognitive symptoms.

In summary, we report for the first time that inbred RHA rats exhibit deficits of non-spatial (i.e., ORT) and spatial (i.e., OLT and DMTP) working/short-term memory, relative to their RLA counterparts. Importantly, the RHA strain also shows impaired long-term memory in the ORT, but only when context habituation is relatively short, whereas longer habituation to the testing context completely rescues such a deficit. This supports the notion that the degree of context habituation may crucially affect performance levels in the ORT, while also suggesting that such context-habituation effects may be partly strain-dependent. Altogether, the present findings add support to the proposal that the inbred RHA rat strain presents a profile of impaired spatial and episodic working memory, and other attentional/cognitive alterations (Fernández-Teruel et al., 2021; Fernández-Teruel et al., 2023; Giorgi et al., 2019; Oliveras et al., 2023), which is compatible with some cognitive phenotypes present in patients with schizophrenia as well as with some symptoms characterizing patients with ADHD.

## Data Availability

The datasets presented in this article are not readily available due to privacy restrictions. Requests to access the datasets should be directed to the corresponding authors.
